# Correction: Vaccination against misinformation: The inoculation technique reduces the continued influence effect

**DOI:** 10.1371/journal.pone.0316311

**Published:** 2024-12-19

**Authors:** The PLOS ONE Editors

## Notice of Republication

This article [1] was republished on December 11, 2024, to address an issue identified post-publication and to provide an updated version of S1 File. Please download this article again to view the correct version.
