# Supplementary material for: Vaccination against misinformation: The inoculation technique reduces the continued influence effect
Source: PLoS One. 2022 Apr 28;17(4):e0267463. doi: 10.1371/journal.pone.0267463 (PMC9049321; doi:10.1371/journal.pone.0267463)
Supplement: S1 File — (DOCX) [file pone.0267463.s002.docx]

All materials were used originally in Polish.

Appendix A. Inoculation procedure

**[Warning]**

Research has shown that people continue to rely on outdated information even when it has been retracted or corrected. An example of this is the fact that some people today still believe that Iraq had weapons of mass destruction even though none have been found. Another example is court proceedings in which jurors continue to use information that has been deemed inadmissible.

In a moment You will be presented with an example of such misleading information within short scenario describing some event that really happened, so all names and objects will be replaced. Please read it carefully and move on.

**[Refutational Preemption]**

[Scenario]

The European Parliament met to approve a new list of food additives. The list included several substances used in food production, including carbohexidine, which biotechnologist Ralf Aschoff have said poses serious health risks, including an increased risk of pancreatic cancer. For this reason, carbohexidine was considered to be withdrawn from the list, but one of the biggest problems with it was the fact that several large food producers used carbohexidine as a preservative. However, several non-EU governments have decided to consider introducing similar restrictions to those proposed by the European Parliament. Due to doubts as to the validity of the use of carbohexidine in food production, a team of scientists from various research centers was appointed to consult. According to them, carbohexidine is not really harmful and can be safely used in food production.

[Belief in misinformation estimate]

Do you think one should be careful when using carbohexidine in food production? Answer on a scale of 0-10 (0 - No, it is safe to use; 10 - Yes, precautions should be taken and it is best to avoid using it).

*Estimate 0-1*

Congratulations, you managed to resist misinformation! It is rare to completely reject it. This is a good decision because the correction came from a highly reliable source, the scientific consensus. In many non-laboratory situations, however, You are certainly exposed to the presence of misinformation and for many reasons you can believe it. The vast majority of people are misinformed in most situations.

*Estimate 2-4*

You've managed to be skeptical about the misinformation, but note that it has hit your point anyway. Although there was only one researcher opposed to the scientific community's consensus, his opinion managed to raise your doubts. However, you are not an isolated incident – the vast majority of people are misinformed in most situations.

*Estimate 5-10*

It seems that the false information about a harmless preservative raised your concerns. Despite the fact that only one researcher opposed the consensus of the scientific community, it managed to raise your doubts. However, you are not an isolated incident - the vast majority of people are misinformed in most situations.

[Follow-up text]

The story presented to You did not actually happen, but it is a reflection of how misinformation can manifest itself. Perhaps You know that situation where a researcher called Wakefield announced that vaccines caused autism. Despite the fact that many scientists from various research centers denied such connections, and Wakefield himself later admitted his mistake, it failed to successfully correct this misinformation. People still believed and still believe in the false cause-and-effect relationship between vaccines and autism, which has led to vaccination refusals, the return of previously eradicated infectious diseases, and enormous financial costs and a serious threat to healthy and vaccinating people. Likewise, people often dismiss information about climate change, even though it is actually happening and is scientifically proven.

While the consequences of belief in the misinformation presented in the story might not be so catastrophic as those mentioned above, note that the mechanisms are the same. Some source first speaks about a certain thing, which is then corrected by the other, highly credible source, i.e. a team of specialists from various research centers - yet people put the opinion of a discredited source equal to or even higher than the opinion of a fully objective, large, highly expert source!

One of the reasons for the persistence of misinformation is the tendency to look for cause-and-effect relationships - when we see that a person vaccinated in childhood displays the characteristics of people on the autism spectrum, we can conclude that it was because the substances in the vaccine contributed to the appearance of such characteristics. So we link one event to another without looking at the facts that provide us with highly reliable sources, such as the scientific consensus.

By disseminating misinformation, we are contributing to the growth of anti-science tendencies, but misinformation is also present in our daily lives - when someone is accused falsely of an act and then it turns out not to be true, people may still believe in the guilt of a falsely accused. Perhaps You have experienced such an unpleasant situation yourself. The fight against misinformation is therefore an extremely important matter, improving both the comfort of everyday life and the prosperity of entire societies.

In a moment, you will read six stories describing certain events. Read them carefully, and then answer a few short questions for each of them. Be vigilant - try not to rely on misinformation if it arise. Be careful when assessing information and try not to be misinformed. Good luck!

Appendix B. Scenarios

**Scenario 1 [Water contamination]**

*Message 1*

The Charleston Water Department has ordered a halt to water intake from the Elk River after numerous reports of fish dying by waterway. The River Elk is the main source of water for Charleston and the surrounding area, providing access to thousands of people. There are no other sources of drinking water in the area.

*Message 2 (Misinformation)*

Meanwhile, the local press announced that the municipal wastewater treatment plant had an accident. The filters and the water drainage pipeline were damaged. Environmental authorities have carried out numerous analyzes of dead fish and plants. It turned out that toxic chemical compounds were detected in them, including high concentration of ammonia, as well as fecal bacteria.

*Message 2 (no-Misinformation control)*

Technicians and chemists joined the rescue operation and stated that the contamination could have occurred as a result of waste of unknown origin entering the river. Environmental authorities have carried out numerous analyzes of dead fish and plants. It turned out that toxic chemical compounds were detected in them, including high concentration of ammonia, as well as fecal bacteria.

*Message 3*

Many people were also admitted to the hospital complaining of symptoms of poisoning, vomiting, severe pain and high fever. Two people died as a result of a serious infection. The regional council assured that the highest priority at the moment is to maintain the best possible quality of drinking water. The town, almost 100 km away, from which fresh water was planned to be delivered in cisterns was asked for help.

*Message 4 (Retraction from Credible source)*

During the investigation, specialists from the national inspection of ecological disasters determined that the failure of municipal wastewater treatment plant did not occur, and that the filters and pipes were completely tight. The timing of the resumption of water intake from the river remains unknown. The city council also plans to run water pipes from another region and replace all pipelines in the city to insure against future similar circumstances.

*Message 4 (Retraction from Expert source)*

Meanwhile, technicians employed at the municipal wastewater treatment plant announced that failure did not occur, and that the filters and pipes were completely tight. The timing of the resumption of water intake from the river remains unknown. The city council also plans to run water pipes from another region and replace all pipelines in the city to insure against future similar circumstances.

*Message 4 (Retraction from Trustworthy source)*

In the meantime, it was announced in the pages of a well-known opinion-forming ecological magazine that the failure of municipal wastewater treatment plant did not occur, and that the filters and pipes were completely tight. The timing of the resumption of water intake from the river remains unknown. The city council also plans to run water pipes from another region and replace all pipelines in the city to insure against future similar circumstances.

**Scenario 2 [Jewellery theft]**

*Message 1*

On May 2, at 3 p.m., the police received a report of the theft of jewelry from the Harter’s house. Gold chains, earrings and pendants with precious stones worth several thousand dollars were stolen.

*Message 2 (Misinformation)*

Mr. and Mrs. Harter discovered they had been robbed as soon as they returned from a week's vacation. The jewelry box was stored in a locked drawer in the dresser in the bedroom. Following further interrogations, it was found that their apartment was visited by the couple's son, Evan, who also did odd jobs for many of the neighbors, to pay off his gambling debts.

*Message 2 (no-Misinformation control)*

Mr. and Mrs. Harter discovered they had been robbed as soon as they returned from a week's vacation. The jewelry box was stored in a locked drawer in the dresser in the bedroom. According to Mrs. Harter's testimony, she was going through the jewellery carefully before the Harters' son, Evan, arrived to take them to the airport.

*Message 3*

Near the bedroom window there was a tall tree, whose branches could be accessed inside the house. However, the police did not find any signs of the break-in. It was also found that several other houses on the same street had been robbed in the last month, but, like the Harter’s house, no signs of break-in have been found and no one has been arrested.

*Message 4 (Retraction from Credible source)*

The police are trying to find out if the Harter’s home is missing other valuables. However, the TV and computer and many other things were not stolen. The theft investigators determined that Evan was out of town and not in the area while the Harters were away.

*Message 4 (Retraction from Expert source)*

The police are trying to find out if the Harter’s home is missing other valuables. However, the TV and computer and many other things were not stolen. Evan's friend testified that Evan was out of town and not in the area while the Harters were away.

*Message 4 (Retraction from Trustworthy source)*

The police are trying to find out if the Harter’s home is missing other valuables. However, the TV and computer and many other things were not stolen. One of the close neighbors, whose house was also recently robbed, testified that Evan was out of town and not in the area while the Harter was away.

*Message 5*

Mr. and Mrs. Harter considered offering a reward for the returning jewelry that had great sentimental value to them. Police are still investigating, trying to link this to previous thefts.

**Scenario 3 [Warehouse fire]**

*Message 1*

On January 25, at 8:58 pm, an alarm signal sounded from the rooms of a stationery warehouse. A major fire was reported in the hall that got out of control and required immediate response.

*Message 2 (Misinformation)*

The alarm was set off by a night security guard who smelled smoke. The fire brigade captain suggested that the fire was triggered by a short circuit in the wiring of a cabinet outside the main warehouse. A police detective found that oil paint cans and high-pressure gas cylinders were reported to be in the closet prior to the fire.

*Message 2 (no-Misinformation control)*

The alarm was set off by a night security guard who smelled smoke. The fire brigade captain suggested that the fire was triggered by a short circuit in the wiring of a cabinet outside the main warehouse. At 4:30 a.m., a police detective announced that the closet was reportedly empty before the fire.

*Message 3*

Firefighters attending the scene reported thick, oily smoke and sheets of ​​flames hindering their efforts, as well as the unusually intense heat that made the fire particularly difficult to control. It has also been learned that a number of explosions occurred during the blaze which seriously endangered firefighters closest to the source of the fire. However, no fatalities have been reported. Two firefighters were also reported to have been taken to hospital as a result of inhaling toxic fumes that had accumulated in the warehouse area.

*Message 4 (Retraction from Credible source)*

In the morning, as a result of a thorough investigation, the detective department announced that the closet that was supposed to contain oil paint cans and gas cylinders was actually empty. The owner of the warehouse estimated that the costs incurred as a result of the damage caused by the fire amounted to hundreds of thousands of zlotys, despite the fact that the warehouse was insured.

*Message 4 (Retraction from Expert source)*

In the morning, the warehouse owner announced that the cupboard that was supposed to contain the oil paint cans and gas cylinders was actually empty. He also estimated that the costs incurred as a result of the damage caused by the fire amounted to hundreds of thousands of zlotys, despite the fact that the warehouse was insured.

*Message 4 (Retraction from Trustworthy source)*

In the morning, the company’s biggest commercial competitor stated that he was convinced that the warehouse had no oil paint cans and gas cylinders, and that the closet had to be empty. The owner of the warehouse estimated that the costs incurred as a result of the damage caused by the fire amounted to hundreds of thousands of zlotys, despite the fact that the warehouse was insured.

**Scenario 4 [Football affair]**

*Message 1 (Misinformation)*

It was announced in the media that Emil Larsson, FC Stockholm star, would not be taking part in the kick-off of the Swedish second division games. A recent medical inspection revealed that Larsson had been under the influence of doping in previous matches.

*Message 1 (no-Misinformation control)*

It was announced in the media that Emil Larsson, FC Stockholm star, would not be taking part in the kick-off of the Swedish second division games. It was reported that Larsson suffered a serious leg injury during his last training session, which may prevent him from playing for months.

*Message 2*

Since the 27-year-old footballer signed with FC Stockholm in 2012, he has become one of the club's best players. He scored 23 goals and 11 assists during the first season. Soon after, he secured his team promotion to a higher league. The press and sports media began to call him "the Swedish Maradona", and Larsson himself did not cease to surprise with the efficiency and endurance, which is remarkable even for a footballer.

*Message 3*

Club chairman Oliver Lindgren, who recently turned down several lucrative Larsson offers, was not contacted. The footballer himself, previously willing to give interviews and posing for photos, began avoiding the media after announcing information in the press. It is speculated that Lucas Johansson, a recent addition to the club, could take the position of Larsson in the opening match against the club's main opponent - Goteborg SK.

*Message 4 (Retraction from Credible source)*

Three days later, Olivier Estevez, director of the International Anti-Doping Committee, announced that Larsson was not involved in the doping affair, and the information was a slander. After a month, it was announced that Larsson had left the club. His further sports career is in question. It is not known what the former footballer plans to do in the future.

*Message 4 (Retraction from Expert source)*

Oliver Lindgren, Larsson's manager, stated that the player was not involved in the doping affair, and the information was a slander. After a month, it was announced that Larsson had left the club. His further sports career is in question. It is not known what the former footballer plans to do in the future.

*Message 4 (Retraction from Trustworthy source)*

Oliver Lindgren, a popular sports commentator, stated that Larsson was not involved in the doping affair, and the information was a slander. After a month, it was announced that Larsson had left the club. His further sports career is in question. It is not known what the former footballer plans to do in the future.

**Scenario 5 [Car accident]**

*Message 1*

On Sunday, the police station received a report about a serious accident involving a bus and a passenger car. The bus hit a steep embankment and rolled over. A passenger car with a dented hood was standing a dozen meters away. Both its driver and passenger survived and were not seriously injured.

*Message 2 (Misinformation)*

The policemen who arrived at the scene undertook a preliminary investigation of the situation. It turned out that over 3 permilles of alcohol was detected in the exhaled air in the driver of a passenger car. The police took the driver on command. Driver seemed stunned, had obvious problems with consistent speech and was moving unsteadily.

*Message 2 (no-Misinformation control)*

The policemen who arrived at the scene examined the drivers of the vehicles with a breathalyzer. It turned out that none of the drivers had any traces of alcohol. The police took the driver on command. Driver seemed stunned, had obvious problems with consistent speech and was moving unsteadily.

*Message 3*

According to the testimonies of witnesses, a passenger car crashed into the bus during an unsuccessful overtaking maneuver. Earlier, it was noticeable that it was not driving straight on the road and ran a red light. One of the witnesses also testified that he saw the driver and the passenger buying cigarettes and bottles of some drink at the gas station. The driver of a passenger car was detained on suspicion of causing a life-threatening road accident. The media speculates that he may face several years in prison.

*Message 4 (Retraction from Credible source)*

A few days later, a team of biochemists found that no traces of alcohol or other psychoactive substances were found in the blood of the passenger car driver. As a result of the accident, over a dozen people were hospitalized, three of which were in a serious condition. It has been estimated that if they survive, their rehabilitation may take months, but even then they may not fully recover.

*Message 4 (Retraction from Expert source)*

A few days later, a passenger in the car, a driver's colleague, told the media that no traces of alcohol or other psychoactive substances were found in the driver's blood. As a result of the accident, over a dozen people were hospitalized, three of which were in a serious condition. It has been estimated that if they survive, their rehabilitation may take months, but even then they may not fully recover.

*Message 4 (Retraction from Trustworthy source)*

A few days later, a local newspaper published an article announcing that no traces of alcohol or other psychoactive substances had been found in the driver's blood. As a result of the accident, over a dozen people were hospitalized, three of which were in a serious condition. It has been estimated that if they survive, their rehabilitation may take months, but even then they may not fully recover.

**Scenario 6 [Political resignation]**

*Message 1*

Minister of Finance Michael Swansen published a short press release in which he stated that he was resigning. Despite numerous questions from journalists, he declined to comment further. Reporters tried to contact a government spokesman to see if they knew anything about why Minister Swansen resigned. Only evasive responses were obtained.

*Message 2 (Misinformation)*

On the same day, it became known that the state prosecutor's office detained Swansen's wife, Anna, the owner of a large retail chain, who was involved in tax evasion and embezzlement of large sums of money. Anna Swansen was one of the most financial supporters of her husband's political campaign. It is believed that mainly thanks to her support, he was given a seat in the government, and then took up the office of Minister of Finance.

*Message 2 (no-Misinformation control)*

On the same day, it was revealed that Swansen's wife, Anna, had died in the hospital of a rare, incurable case of cancer. Anna Swansen was one of the most financial supporters of her husband's political campaign. It is believed that mainly thanks to her support, he was given a seat in the government, and then took up the office of Minister of Finance.

*Message 3*

Some of Michael Swansen's associates saw the former minister packing hasty boxes in his office and destroying documents on the day of his departure. Afterwards, he left the Ministry headquarters without a word and went home. It is suspected that in the current situation there may be serious changes in the ministry. Reporters surrounded Swansen's house and waited for him to appear, but were unable to speak to him.

*Message 4 (Retraction from Credible source)*

Three days after the events, the UK Anti-Corruption Bureau announced that Anna Swansen was not involved in tax fraud. Journalists informed the public that Michael Swansen has left the capital and will not comment on his resignation. The office of the minister of finance was taken over by the previous deputy minister.

*Message 4 (Retraction from Expert source)*

Three days after the events, Michael Swansen told the media that his wife was not involved in tax fraud. Journalists informed the public that Michael Swansen has left the capital and will not comment on his resignation. The office of the minister of finance was taken over by the previous deputy minister.

*Message 4 (Retraction from Trustworthy source)*

Three days after the events, the well-known and respected journalist and political critic Jacob Miller stated that Anna Swansen was not involved in tax fraud. Journalists informed the public that Michael Swansen has left the capital and will not comment on his resignation. The office of the minister of finance was taken over by the previous deputy minister.

Appendix C. Test

**[Inference questions]**

Scenario 1 [Water contamination]

1. Where could the toxic substances that poison the river have come from?
2. Who should police investigators interrogate about river poisoning with special attention?
3. What could have been the causes of poisoning among the people who presented to the hospital.
4. What should be done to avoid similar incidents in the future?
5. Are there reasons to punish any person or institution for poisoning?

Scenario 2 [Jewellery theft]

1. Why wasn't the TV stolen?
2. How could a thief get into the house?
3. How do you think a thief got into a locked drawer to steal a jewellery box?
4. Who could be responsible for other thefts in the area recently?
5. Who, if anyone, should be questioned more thoroughly by the police?

Scenario 3 [Warehouse fire]

1. Why did the fire spread so quickly?
2. What was the possible cause of the toxic fumes?
3. What was the relevance of the closed for the fire?
4. On what aspect of the fire may the police want to continue investigation?
5. What could have caused the explosions?

Scenario 4 [Football affair]

1. Why did Emil Larsson decide to leave the club?
2. What could be behind Larsson's extraordinary sports performance?
3. Why did Larsson who previously did not shy away from the media started avoiding them?
4. What should be done to avoid similar incidents in the future?
5. Should Larsson be suspended before he quit the club himself? If so, why?

Scenario 5 [Car accident]

1. For what reason could the driver of a passenger car have problems with speech?
2. Why was the driver of the passenger car unsteadily moving?
3. What could be the reason why the passenger car did not move straight on the road?
4. Why did the driver of a passenger car run a red light?
5. What kind of drink could be in the bottles that the car driver and his colleague were buying at the station?

Scenario 6 [Political resignation]

1. Why could Michael Swansen step down as minister?
2. What could the documents Swansen destroyed before leaving the office be about?
3. Why did Michael Swansen avoid talking to the media and refuse to explain himself because of his resignation?
4. Why was Swansen leave city?
5. Are there reasons to believe that Swansen was not an honest politician? If so, what?

**[Belief scales]**

Scenario 1 [Water contamination]

*Belief in misinformation*

To what extent do you believe that pollution from municipal wastewater treatment plant is responsible for the contamination of the Elk River?

*Belief in retraction (C)*

To what extent do you believe in the opinion of specialists from the national inspection for environmental disasters that the failure in the municipal wastewater treatment plant did not occur?

*Belief in retraction (E)*

To what extent do you believe in the opinion of the municipal wastewater treatment plant employees that there was no accident in their company?

*Belief in retraction (T)*

To what extent do you believe in the opinion of a well-known opinion-forming ecological magazine that the failure in the municipal wastewater treatment plant did not occur?

Scenario 2 [Jewellery theft]

*Belief in misinformation*

To what extent do you believe that Evan, son of the Harters, could have committed the theft?

*Belief in retraction (C)*

To what extent do you believe the theft investigators who say that Evan, the son of Harters, was out of town at the time of the theft?

*Belief in retraction (E)*

To what extent do you believe in Evan friend’s opinion that Mr. and Mrs. Harter's son was at the time of the robbery out of town?

*Belief in retraction (T)*

To what extent do you believe in the opinion of Harter's neighbor, who was also robbed, that Evan was outside the town at the time of the robbery?

Scenario 3 [Warehouse fire]

*Belief in misinformation*

To what extent do you believe that oil paints and gas cylinders stored in a closet are responsible for the violent outbreak of a fire?

*Belief in retraction (C)*

To what extent do you believe the regional fire and safety inspector said that there was no gas and paint cylinder in the closet and that it was empty?

*Belief in retraction (E)*

To what extent do you believe the warehouse owner said that there was no gas and paint cylinder in the closet and that it was empty?

*Belief in retraction (T)*

To what extent do you believe the company's commercial competitor stated that there was no gas and paint cylinder in the closet and that it was empty?

Scenario 4 [Football affair]

*Belief in misinformation*

To what extent do you believe that Emil Larsson left FC Stockholm because of the doping scandal?

*Belief in retraction (C)*

To what extent do you believe in the opinion of Olivier Estevez, director of the International Anti-Doping Committee, that Emil Larsson was not involved in the doping issue?

*Belief in retraction (E)*

To what extent do you believe in the opinion of Olivier Lindgren, manager of Emil Larsson, that the footballer was not involved in the doping issue?

*Belief in retraction (T)*

To what extent do you believe the opinion of Olivier Lindgren, a popular and respected sports commentator, that Emil Larsson was not involved in the doping case?

Scenario 5 [Car accident]

*Belief in misinformation*

To what extent do you believe that the driver of a passenger car was driving under the influence of alcohol?

*Belief in retraction (C)*

To what extent do you believe in the opinion of biochemists and toxicologists that the driver had no alcohol or other psychoactive substances in his blood while driving?

*Belief in retraction (E)*

To what extent do you believe in the opinion of the passenger of the car, a colleague of the driver, that the driver was not drunk while driving?

*Belief in retraction (T)*

To what extent do you believe the local newspaper article saying that the driver was not drunk while driving?

Scenario 6 [Political resignation]

*Belief in misinformation*

To what extent do you believe that Minister of Finance Michael Swansen has resigned due to tax fraud?

*Belief in retraction (C)*

To what extent do you believe in the opinion of the UK Anti-Corruption Bureau that Anna Swansen, Michael Swansen's wife, was not involved in tax fraud?

*Belief in retraction (E)*

To what extent do you believe in Michael Swansen's view that his wife was not involved in tax fraud?

*Belief in retraction (T)*

To what extent do you believe in the opinion of Jacob Miller, a well-known political critic, that Anna Swansen was not involved in tax fraud?

Appendix D. Variants of scenario configurations

|  | **S 1** | **S 2** | **S 3** | **S 4** | **S 5** | **S 6** |
| --- | --- | --- | --- | --- | --- | --- |
| **1** | credible | noMisinf | noRetr | trust | expert | noMisinf |
| **2** | noMisinf | credible | noMisinf | noRetr | trust | expert |
| **3** | expert | noMisinf | credible | noMisinf | noRetr | trust |
| **4** | trust | expert | noMisinf | credible | noMisinf | noRetr |
| **5** | noRetr | trust | expert | noMisinf | credible | noMisinf |
| **6** | noMisinf | noRetr | trust | expert | noMisinf | credible |
