# Supplementary material for: Vaccination against misinformation: The inoculation technique reduces the continued influence effect
Source: PLoS One. 2022 Apr 28;17(4):e0267463. doi: 10.1371/journal.pone.0267463 (PMC9049321; doi:10.1371/journal.pone.0267463)
Supplement: Attachment — Submitted filename: Response to reviewers.doc [file pone.0267463.s003.doc]

Prof. Romuald Polczyk

Institute of Psychology, Jagiellonian University

6 Romana Ingardena Stret, 30-060 Kraków, Poland

Tel. (+48) 12 663 24 36

Email: romuald.polczyk@uj.edu.pl

RE: Revised version of the article: “Vaccination against misinformation: The inoculation technique reduces the continued influence effect”

Dear Editors,

Please find enclosed the revised version of our article. Below we listed all changes that were made:

Major changes:

1. We shortened the discussion part by about 1/3 of its previous length and made it clearer.
2. We corrected some linguistic mistakes thorough the text.
3. Aside from the research mentioned by reviewers, we added in the introduction and in the discussion sections some new research references:
   a. <https://doi.org/10.3758/s13421-021-01232-8>
   b. <https://doi.org/10.3758/s13421-011-0179-8>
   c. <https://doi.org/10.1073/pnas.2020043118>, <https://doi.org/10.1111/jcom.12171>
   d. <https://doi.org/10.1080/01296612.2017.1384145>
   e. <https://doi.org/10.1080/17437199.2010.521684>
   f. Connor Desai SA. (Dis) continuing the continued influence effect of misinformation [Doctoral thesis]. [London]: City, University of London; 2018.
   g. Connor Desai SA, Reimers S. Some misinformation is more easily countered: An experiment on the continued influence effect. In: Proceedings of the 40th Annual Meeting of the Cognitive Science Society. Austin, TX: Cognitive Science Society; 2018. p. 1542–7.

We also tried to attend to all comments and recommendations made by the reviewers. Here is the list of the specific changes and corrections:

**Reviewer A**:

1. We corrected the fragment “new highly applicable technique has been discovered” to “certain boundary conditions for inoculation efficiency have been discovered” (p. 2).
2. We added a reference to the research mentioned and also <https://doi.org/10.1186/s41235-020-00241-6> when it comes to failure to replicate Nyhan & Reifler’s (2010) study (p. 3).
3. We added references mentioned about the KReC model to the discussion of the integration accounts (p. 4).
4. We rephrased the sentence „it is assumed that the model updating leads to local consistency and to errors in drawing conclusions when the situation is assessed globally” into „In the case of CIE, it is assumed that the model updating leads to local consistency but conclusions are drawn globally, leading to errors due to local sustaining of misinformation” (p. 4).
5. We rephrase some phrases in the description of the selective retrieval account to make it clear that this approach does not necessarily require the assumption of dual processes and that the CIE could also occur when the correction is not activated (p. 4 / p. 5).
6. We have deleted the fragment „based on cognitive resources” from “controlled processes based on cognitive resources” (p. 5).
7. We corrected the description of the illusory truth effect (p.5)
8. Aside from mentioned <https://doi.org/10.1098/rstb.2020.0145>, we added also <https://doi.org/10.1098/rsos.180593>, <https://doi.org/10.1098/rsos.160802>, <https://doi.org/10.1111/pops.12586>, when it comes to discussing the role of worldview effects to make the discussion more balanced (p. 5).
9. We mentioned <https://doi.org/10.1371/journal.pone.0210746> to the discussion of factors useful to reduce misinformation reliance (p. 6).
10. We change some phrases about the fragment where techniques of limiting the CIE were discussed, that previously claimed that „no novel techniques limiting the impact of misinformation in the CIE paradigm were identified”. We meant the particular laboratory „narrative paradigm” of CIE, as it is usually performed (Ecker et al., 2010; Johnson and Seifert, 1994) and as it was defined by Connor Desai (2018) in her work. Nevertheless, we added also some clarification about it when discussing Tay et al.’s (2021) study (p. 9).
11. We mentioned that in the Cook et al.’s (2017) study the inoculation led to the generalization of resistance from the explanation of strategy used by the tobacco industry to misinformation about climate change (p. 8).
12. We discussed Tay et al.’s (2021) 10.31234/[osf.io/48zqn](http://osf.io/48zqn) work about the inoculation against fictional misinformation (p. 9). We also refer to this study in the discussion when it comes to failing to replicate Rich & Zaragoza’s implied vs. explicit misinformation difference (p. 22) and in the discussion about the link between misinformation compliance at cognitive and behavioral levels (p. 29).
13. We repeated the power analysis in the G*Power, but we achieved different results than You did. Perhaps it happened because You changed the content of the field „Number of groups” to the field „Number of measurements”. Note that in our study groups were 2 and measurements were 5. We attach a screenshot of our calculations below.

14. We added clarification about the rating scales. The rating scales concerned only the 3 retraction conditions. Each participant had access to only one scenario for each retraction condition, so the scales were for only three scenarios. As there were 2 scales for each scenario, 6 scales per person are obtained (p. 14).
15. We added changes when it comes to the examples of the participants’ responses – now we stick only to two scenarios (warehouse fire and car accident) (p. 14 and throughout).
16. We deleted the „one-way” from the ANOVA description (p. 16).
17. We rephrase „main effect of the interaction” to „interaction effect” (p. 16).
18. We changed Figure 1 that the no-correction control condition is now presented on the left (we also made appropriate changes to the description of the figure) and specified error bars in the descriptions of figures. We also found out that the issues with y-axis labels were due to the conversion of the files from the .cdr to .eps. We couldn't fix this in the EPS file, so we're sending the corrected figures in a PDF version.
19. We corrected errors resulting from the translation from Polish to English, e.g. exaggerated language (throughout the text).
20. We added a reference to the Brydges et al.’s (2020) <https://doi.org/10.1080/20445911.2020.1849226> study when it comes to the finding that belief in misinformation predicted the reliance on misinformation (p. 23).
21. We rephrased and added clarification when it comes to speculations about skepticism as a result of maintaining the coherence of the mental model, also adding a reference to the motivational factors (<https://doi.org/10.3758/s13421-021-01232-8>) (p. 21).
22. We did our best but unfortunately we couldn’t find any research concerning both response criterion effects and skepticism.
23. In the response to the comment about why „it could be considered “right to rely on critical information” in the expert-correction condition?”: we have deleted the fragment (p. 22) while shortening the discussion part, but in the latter text (p. 24) there is an explanation of that reasoning with the reference to the Connor Desai et al.’s (2020) study, in which authors concluded that under certain conditions (e.g. when the source of the retraction is unreliable) it may be considered rational to rely on misinformation. In this study, the expert source of the retraction was operationalized as in Guillory & Geraci’s (2013) study, and therefore it could be considered by participants as unreliable - thus it may be rational to rely on critical information.
24. We rephrased the part about generated information to be more clear (p. 22).
25. We deleted the fragment about the repetition of misinformation in the context of a correction that may lead to increased misinformation reliance (previously on p. 24).
26. We deleted the fragment „inoculation does not immunize to the scenario schema” as it was not clear and redundant, and also in the process of shortening the discussion section (previously on p. 24).
27. We added a note that two theoretical CIE accounts are not exclusive and may be complementary both in the introduction (p. 3) and in the discussion (p. 25).
28. We corrected some sentences mentioned:
    a. “The results showed that the reliability of the sources of corrections did not affect their processing when participants were not inoculated, but, at the same time, a significant reduction in the reliance on misinformation among vaccinated participants when the correction was made from a highly credible source was observed.” was broke down into: „The results showed that the reliability of the sources of corrections did not affect their processing when participants were not inoculated. However, under inoculation condition, a significant reduction in the reliance on misinformation among vaccinated participants when the correction was made from a highly credible source was observed.” (p. 2).
    b. We added clarification what “within the *remembering framework*” means by adding to it sentence: “within the *remembering framework* describing the conversion from memory traces to behavioral memory statements” (p. 2).
    c. The sentence “where the whole model is reconstructed and new is created” was changed to „where the whole model is reconstructed and a new one is created” (p. 4).
    d. The sentence “information being coded is always treated as it was true and may be falsified later by attaching a negation tag to itself” was changed into “information being encoded is always treated initially as if it were true and is only falsified subsequently by attaching a negation tag” (p. 5).
    e. The sentence “non-memory factors, e.g. motivational (e.g. attitudes and the worldview)” was changed into “non-memory factors such as motivational factors (e.g., person’s attitudes and worldview)” (p. 5).
    f. We rephrased sentence containing “attitudes that are not vaccinated” to the „Inoculation treatments are also able to protect attitudes that were not the subject of protection by vaccination but are somewhat related to the target attitude” (p. 7).
    g. The sentence “Given that retractions are more effective if their source is trusted but not necessarily expert, not only replication of existing reports can be expected (Hypothesis 1), but also different vaccination effects depending on the reliability of the sources of the retractions.” was changed into „Given that retractions are more effective if their source is trusted but not necessarily expert (16,19,50), replication of existing reports can be expected (Hypothesis 1). We also expected different vaccination effects depending on the reliability of the sources of the retractions.” (p. 10).
    h. The fragment “For ratings 0 or 1, message informing of surviving the misinformation attack was presented; for ratings 2-4 it informed about the occurrence of resistance, but the impact of misinformation on the participant’s decision was accented” was rephrased into „For ratings 0 or 1 message informed participants about their low misinformation compliance. For ratings 2-4 message informed participants about their medium misinformation compliance: it accented to both occurrence of some resistance and some acceptance of misinformation” (p. 15).
    i. We corrected a mistake in the sentence “The effect of inoculation on belief in misinformation and retraction, as hypothesized, was observed for the credible source condition, *where~~as~~* inoculation significantly increased belief in retraction and lowered belief in misinformation” (p. 19).
29. As possible, due to comments, we tried to simplify sentence structures, specified variables when referring to them, and unified terms used in the discussion section.
30. We corrected a typo „Huyhn” into „Huynh” (p. 16).

# Reviewer B

1. We added reference to the research on cynicism and decreasing trust in the accurate news as a result of the inoculation procedure (<https://doi.org/10.1080/15205436.2018.1511807>, <https://doi.org/10.1080/08838151.2016.1273923>) in the discussion section (p. 22).

Yours sincerely

Romuald Polczyk
