# Supplementary material for: Vaccination against misinformation: The inoculation technique reduces the continued influence effect
Source: PLoS One. 2022 Apr 28;17(4):e0267463. doi: 10.1371/journal.pone.0267463 (PMC9049321; doi:10.1371/journal.pone.0267463)
Supplement: Attachment — Submitted filename: Response to reviewers - 2nd review.doc [file pone.0267463.s004.doc]

Prof. Romuald Polczyk

Institute of Psychology, Jagiellonian University

6 Romana Ingardena Street, 30-060 Kraków, Poland

Tel. (+48) 12 663 24 36

Email: romuald.polczyk@uj.edu.pl

RE: Revised version of the article: “Vaccination against misinformation: The inoculation technique reduces the continued influence effect”

Dear Editors,

Please find enclosed the revised version of our article. Below we list all changes that have been made:

Major changes:

1. We have added a paragraph about the source characteristics and their relation to the CIE and inoculation mechanisms (p. 10/11).
2. We have rebuilt the hypothesis section, linking it to the newly added paragraph mentioned above (p. 11/12) and adding many more explanations.
3. We have added a new “Power analysis” section, in which we have reworked the fragment about power analysis (p. 13). We also have added a fragment about the limitations of achieved power in the discussion (p. 33).
4. We have expanded the fragment about the practical implications of our results in improving inoculation interventions (p. 32/33).
5. We have corrected some linguistic mistakes thorough the text.
6. Aside from the research mentioned by reviewers, we have added some new research references in the introduction and in the discussion sections:
   a. <https://doi.org/10.1111/j.1475-682X.2009.00280.x>
   b. <https://doi.org/10.1080/10463280802643640>
   c. <https://doi.org/10.1111/j.1559-1816.2010.00620.x>
   d. <https://doi.org/10.1016/j.cognition.2018.06.011>
   e. <https://doi.org/10.1038/s44159-021-00006-y>
   f. Szpitalak, M. (2015). *W kierunku poprawy jakości zeznań świadków. Pozytywne i negatywne następstwa ostrzegania o dezinformacji*. Wydawnictwo Uniwersytetu Jagiellońskiego.

We have also tried to address to all comments and recommendations made by the reviewers. Here is the list of the specific changes and corrections:

**Reviewer 1**:

1. We have added a new “Power analysis” section in which we have reworked the fragment about power analysis (p. 13). We hope that it is more adequate now. Also, we have added a fragment about the limitations of achieved power in the discussion (p. 33).
2. We have changed “behavioral memory statements” to “behavioral manifestations of memory” (p. 2, Abstract).
3. Unfortunately, we did not find any oddities in the format of “Guillory & Geraci”, therefore we did not make any corrections.
4. We have corrected “most likely be an effect” to “most likely an effect” (p. 24).
5. We have corrected “as was in … condition” to “as was the case in the … condition” (p. 25).
6. We have corrected:
   a. “only when one generates … but not when they are” to “only when one generates … but not when one is” (p. 25);
   b. “one may ... with their worldview” to “one may ... with one’s worldview” (p. 28);
   c. “If one experiences … they may want to … keep their assumptions” to “If one experiences … one may want to … keep one’s assumptions” (p. 29);
   d. “one can … they declare that they don’t believe” to “one can…one declares that one does not believe” (p. 30).
7. We have corrected “not exclusively for the previous explanation” to “not mutually exclusive with the previous explanation” (p. 27).
8. We corrected “a crucial role may play a number of factors” to “a number of factors may play a crucial role” (p. 28).
9. We have corrected:
   a. “yet take advantage of misinformation” to “yet make use of misinformation” (p. 29);
   b. “failure to the retraction retrieval” to “failure of retraction retrieval” (p. 29).
10. We have corrected “by, for example, by” to “for example, by”.
11. We have added <https://doi.org/10.1111/j.1475-682X.2009.00280.x> to the discussion, as suggested (p. 30).
12. We have corrected “exposed to some problem-solving process” to “involved in some problem-solving process” (p. 30).
13. We have rephrased “under retractions’ highly credible source condition” to “under conditions where a retraction came from a highly credible source” (p. 32).
14. We have changed “inoculation efficacy” to “inoculation effectiveness” (p. 33).
15. We have corrected “exploring the inoculation” to “exploring inoculation” (p. 34).
16. We have corrected “impact of inoculation on misinformation” to “impact of inoculation on misinformation reliance” (p. 34).

# Reviewer 3

1. We have added a paragraph about the source characteristics and their relation to the CIE and inoculation mechanisms (p. 10/11).
2. We have rebuilt the hypothesis section, linking it to the newly added paragraph mentioned above (p. 11/12) and adding much more explanations. We hope that the hypotheses are now more understandable.
3. We have added a table with examples of certain sources from one of the scenarios (p. 15).
4. Unfortunately, it is true that we have no way of objectively assessing whether and to what extent may be the differences between the same source conditions in different scenarios. The best way to standardize these examples would be to perform a pilot study; however, for obvious reasons, we cannot do this. Thus, we made the decision to cover this issue on p. 33.
   Also, Scenario 4 in the Appendix seems to be complete (the scenario was taken from Ecker & Antonio (2021), where it starts similarly).
5. We have added references to the specific hypotheses in the Results sections
   a. H1 – p. 19
   b. H2 – p. 19
   c. H3 – p. 19
   d. H4 – p. 20/21
   e. H5 – p. 21/22
6. We have expanded the fragment about the practical implications of our results in improving inoculation interventions (p. 32/33). Although we could not add the proposed text, because our study did not include analytical thinking, we added it on p. 33.
7. Although we could not add the proposed Pennycook and Rand text in this excerpt, because our study did not include analytical thinking, we have added it to p. 32/33.

Yours sincerely

Romuald Polczyk
